# Emergency care visits at a South African hospital: Implications for healthcare services and policy

**DOI:** 10.4102/safp.v66i1.5816

**Published:** 2024-03-18

**Authors:** Jacob A. Adewole, John M. Tumbo, Henry I. Okonta

**Affiliations:** 1 Department of Family Medicine and Primary Health Care, Faculty of Medicine, Sefako Makgatho Health Sciences University, Pretoria, South Africa; 2 Brits District Hospital, Bojanala District, Rustenburg, South Africa; 3 Health Services Department, Bojanala District, Rustenburg, South Africa

**Keywords:** accident and emergency, triage, district hospital, visits, acuity, disposal

## Abstract

**Background:**

A robust knowledge on the pattern of use of emergency care resources not only serves as an indicator of universal access to care but also provides a basis for quality improvement within the health system. This study was undertaken to describe the pattern of emergency room visits at Brits District Hospital (BDH) in North West province, South Africa. The objectives of this study were to determine the sociodemographic characteristics of emergency department (ED) users and other patterns of ED use.

**Methods:**

This was a cross-sectional descriptive study that was conducted at a district hospital. All patients who reported for emergency care in the ED in 2016 were eligible for the study. Data were extracted and analysed from a systematic sample of 355 clinical notes and hospital administrative records.

**Results:**

The age group that visited the ED most frequently (25.3%) was 25–34 years old. A high proportion of the ED users (60%) were self-referred, and only 38% were transported by the emergency medical response services (EMRS). Few (5.6%) presentations were of a non-urgent nature. Trauma-related conditions accounted for the most frequent presentation at the ED (36.5%).

**Conclusion:**

Although most ED users were self-referred, their clinical presentations were appropriate and underscore the need for policy strategies to reduce the burden of trauma in the catchment population

**Contribution:**

The study findings may have an impact on future health policies by providing decision-makers with baseline information on the pattern of use of ED resources, ensuring better resource deployment and greater access to care.

## Introduction

Understanding the pattern of emergency care services utilisation has become a global imperative. Policy makers and managers of healthcare systems require information on the demographic characteristics of the users of healthcare facilities and the local burden of diseases for proper structuring and resource allocation of the healthcare system.^1^

The structure of the South African public health system remains hierarchical from the mobile health units and primary healthcare facilities as the entry points, with upward referral through district hospitals, secondary level hospitals and eventually tertiary hospitals. The cost and complexity of care also increase along similar lines.

Emergency department (ED) visits at both the general (district) and specialised (secondary and tertiary) hospitals impact significantly on the cost and quality of care. This is a result of the ever-increasing number of patients seeking care who are bypassing the lower levels of care. In many instances, the health problems prompting hospital ED visits are simple, amenable to ambulatory care and manageable at the primary care level, often described as ambulatory care-sensitive conditions (ACSCs). Emergency departments are also viewed by patients with non-urgent clinical presentations as providing a better and quicker access to the healthcare system. The treatment of ACSCs at primary care clinics rather than hospital EDs is associated with significant cost savings.^2^

Issues of lengthy ED waiting times and overcrowding provide an indication of access and quality of healthcare.^1,3^ The recent application of machine learning algorithms for the optimisation of emergency services also requires background data on the pattern of ED use.^4^ Availability of information on the pattern of ED use is also important because of the highly variable nature of an ED’s case mix, especially at the primary care level.^5^

Considerable research has been done on the pattern of ED use, but the outcomes of such studies tend to demonstrate region-specific trends that are not generalisable. Little is known about the pattern of use of emergency services at BDH, and consequently, Madibeng Sub-District in the North West province of South Africa. The aim of this study was to determine the pattern of visits to the ED at the Brits District Hospital (BDH) with the expectation that the outcome of this study would assist policy makers, health facility managers and public health practitioners in the North West province to define and strengthen emergency services.

## Research methods and design

This was a cross-sectional descriptive study based on the analysis of hospital administrative records and the clinical notes of patients seen at the emergency unit at BDH from 01 January 2016 to 31 December 2016.

The Brits District Hospital is a 190-bed, medium-sized district public hospital situated in Brits town in the North West province of South Africa that serves a population of 570 000 spread out in the 3900 square kilometres land space. The hospital is manned by 643 clinical and non-clinical staff of whom 44 and 401 are employed as doctors and nurses, respectively. Brits District Hospital is a referral hospital for both private and public primary care facilities within its boundaries and adjoining communities.

All patients who reported for emergency care in the ED during the study period were eligible for our study. The calculated sample of 362 was extracted from the sampling frame of 19 560 visits. Data were extracted from a systematic sample of 355 clinical notes and hospital administrative records by trained data capturers. Daily visits to the hospital were divided into six clusters of 4 h each. Every third patient record was drawn up as a systematic sample from the relevant daily cluster. Clusters were sampled in sequence. Extraction of de-identified data was made from the clinical records of patients and the emergency unit patients’ attendance register by two trained data capturers using a standardised piloted 22-point data collection tool. Data were collected on each patient’s age, sex, employment status, residential address, arrival and departure time in the emergency room, date and day of emergency room visit, time taken to be seen by the doctor and total time spent in the ED, referral pattern, mode of transport to hospital, acuity score, presenting complaints and diagnosis made during consultation and mode of disposition following consultation. The ‘estimated distance from BDH’ was obtained from Google Maps by the investigator as the shortest distance on a motorable route between the patient’s residential area and BDH.

The data collected from the study were entered and stored in an encrypted, password-protected Microsoft Excel (Microsoft, Redmond, Washington, United States [US]) file. Data were imported as string variables into the Statistical Package for the Social Sciences (SPSS) version 25.0 (IBM, Chicago, Illinois, US) file for further analysis. Study results were presented mainly as descriptive statistics. Tables and graphs were created using Excel. Chi-square goodness-of-fit test was used to make a comparison between hypothesised population distribution and the diagnostic sample population distribution. Bivariate analysis was carried out to evaluate the relationship between certain variables.

### Ethical considerations

Approval for the study was granted by the Research Ethics Committee of Sefako Makgatho Health Sciences University, South Africa (SMUREC/M/12/2018:PG).

## Results

### Demographic characteristics

The sex distribution of patients visiting the ED (Table 1) shows a predominance of male ED users (51.3%). The age group that visited the ED most frequently was 25–34 years (25.3%, *n* = 90). Most of the patients in the age range 15–64 years self-reported that they were unemployed (62.7%).

**TABLE 1:** Demographic characteristics of emergency department users (*n* = 355).

Category	Frequency	Percentage (%)
**Sex**		
Female	173	48.7
Male	182	51.3
**Age (years)**		
< 5	34	9.6
5–14	26	7.3
15–24	58	16.3
25–34	90	25.3
35–44	72	20.3
45–54	29	8.2
55–64	25	7.0
> 65	18	5.1
No response	3	0.9
**Employment (15–64 years)**		
Employed	104	37.3
Unemployed	175	62.7
**Estimated distance (km)**		
0–5	122	34.4
6–10	10	2.8
11–20	71	20.0
21–30	87	24.5
> 30	63	17.7
No response	2	0.6

Private vehicle use (57.1%) was the commonest transportation mode employed by patients presenting at the ED, followed by the EMRS (37.7%) at a considerably lower proportion (Table 2). Only a small proportion of these ED users were brought by police officials (2%) and hired taxi/vehicle (1.7%). Interestingly, two patients (0.6%) were able to walk from home to the ED. None of the ED users reported engaging in the services of a private ambulance.

**TABLE 2:** Distribution of patients by transport, referral and triage patterns.

Category	Frequency	Percentage (%)
**Transport**		
Walking	2	0.6
Private vehicle	203	57.1
Hired a taxi	6	1.7
EMRS	134	37.7
Private ambulance	0	0.0
State/police	7	2.0
No response	3	0.9
**Referral**		
Self-referred	213	60.0
Private practitioner	19	5.3
Public facility/clinic	114	32.1
State/police	6	1.7
No response	3	0.9
**Triage**		
Red (immediate)	8	2.3
Orange (very urgent)	83	23.4
Yellow (urgent)	238	67.0
Green (routine)	20	5.6
Blue (dead)	0	0.0
No response	6	1.7

EMRS, emergency medical response services.

A total of 60% of patients visiting the ED for emergency care were self-referred (Table 2). About one-third (32.1%) of the ED users were referred from other public facilities and clinics. Only a small proportion of patients were referred from private practitioners (5.3%) and law enforcement agents (1.7%). Patient triage categories were represented as Red for ‘immediate care’ (2.3%), Orange for ‘very urgent’ (23.4%), Yellow for ‘urgent’ (67%) and Green for ‘routine’ (5.6%). None of the patients arrived dead (Blue) at the ED (Table 2).

The highest proportion of self-referred patients by level of acuity was found in the ‘routine’ triage category (65%) (Table 3).

**TABLE 3:** Patient referral patterns and triage category.

Triage	Frequency	Self-referred	Proportion self-referred (%)
Red (immediate care)	8	4	50.0
Orange (very urgent)	83	48	57.8
Yellow (urgent)	238	145	60.9
Green (routine)	20	13	65.0

**Total**	**349**	**210**	**60.2**

The patient diagnostic classifications (Table 4) reveal that trauma-related conditions were by far the most frequent clinical conditions seen at the ED (36.5%). Other leading diagnostic classifications among ED users include respiratory conditions (9.5%), gastrointestinal illnesses (9.5%), genitourinary conditions (8.4%), ‘ear, nose and throat conditions (ENT)’ (5.3%) and ‘poisoning and intoxication’ (5.1%). All forms of trauma occurred most frequently in the age range 15–54 years.

**TABLE 4:** Distribution of patients by diagnostic classification.

Category	Frequency	Percentage (%)
Trauma-related	157	36.5
Respiratory system	41	9.5
Gastrointestinal tract	41	9.5
Genitourinary system	36	8.4
ENT	23	5.3
Poisoning and intoxication	22	5.1
Metabolic disorders	17	3.9
Other infections	15	3.5
Cardiovascular system	14	3.3
HIV-related	11	2.6
Nervous system	11	2.6
Psychiatric illnesses	10	2.3
Skin	9	2.1
Ophthalmic disorders	9	2.1
TB-related	5	1.2
Others	5	1.2
Musculoskeletal disorders†	4	0.9

**Total**	**430** ‡	**100.0**

ENT, ear, nose and throat; TB, tuberculosis; ED, emergency department.

† , Musculoskeletal disorders, excluding trauma-related causes.

‡ , Some ED users had multiple diagnostic classifications.

Most ED users spent between 1 h and 3 h (32.4%) in the unit. Only eight patients (2.3%) spent more than a day in the ED (Table 5).

**TABLE 5:** Total time spent by patients in the emergency department.

Total time spent in the ED	Frequency	Percentage (%)
Less than 1 h	52	15.0
1–2 h 59 min	112	32.4
3–5 h 59 min	75	21.7
6–11 h 59 min	67	19.4
12–24 h	32	9.2
1–2 days	7	2.0
More than 2 days	1	0.3

**Total**	**346**	**100.0**

ED, emergency department.

A total of 70% of patients seen at the ED were treated and discharged (Table 6), and 14.5% of the patients were admitted into the wards after initial treatment at the ED. The proportion of ED users referred to level two and academic hospital for specialised care was 6.4%. Three patients were deemed appropriate for ambulatory care, one at the clinic (0.3%) and two at the outpatient department (0.6%). All three patients were down-referred accordingly. Two patients died in the ED (0.6%) and eight left without being seen (2.3%).

**TABLE 6:** Patient treatment outcomes.

Treatment outcome	Frequency	Percentage (%)
Treated and discharged	264	75.0
Admitted into the ward	51	14.5
Transferred to higher level of care	23	6.4
Left before being attended to	8	2.3
Assessed and booked for OPD	2	0.6
Died in ED	2	0.6
Assessed and down-referred	1	0.3
Refused hospital treatment	1	0.3

**Total**	**352**	**100.0**

ED, emergency department; OPD, outpatient department.

## Discussion

Emergency department users at BDH are predominantly male, often aged 15–44 years and report being unemployed. The sex distribution of patients visiting the ED compares favourably with the sex distribution of the host community, with males (53%) and females (47%).^6^ This is also consistent with findings obtained from studies in EDs with high proportions of trauma-related conditions as part of their case mix. However, our study revealed a much higher unemployment rate (63%) than that reported by Statistics South Africa (30.4%).^6^ The employment status of ED users below 15 years and above 64 years was not investigated as these individuals were regarded as minors and pensioners, respectively.

In a nationwide study across EDs in Kenya, two peak age categories were observed, 0–9 years (27%) and 20–29 years (25%), in contrast to the findings at Princess Marina Hospital in Gaborone, Botswana, in which almost half of the ED users (49.1%) were within the age range of 25–49 years.^7,8^ The wide variation in age pattern of ED users across regions is further confirmed by the report of a systematic review of emergency care in low- and middle-income countries, which puts the median age of ED users at 35 years (interquartile range [IQR]: 6.9–41.0).^9^ However, at AL-Yarmook Urgent Care Centre in Riyadh, Saudi Arabia, Arafat and colleagues found a much younger peak, with half of the ED users being below the age of 15 years.^10^ Our study revealed a peak prevalence between 25 years and 34 years (25.5% of ED users) and indicated a greater use of ED resources by young to early middle-aged adults (15–44 years). Statistics South Africa puts the proportion of the population below 15 years at 29.4%.^6^ This is higher than the proportion of ED users in the same age category (17.1%).

Trauma-related conditions make up the highest proportion of the case mix found among ED users at BDH. The male predominance in the use of ED in this study is in contrast with the results from a study in Bloemfontein, which showed that more females (57.7%) used the ED.^11^ They attributed this difference to the presence of a dedicated secondary trauma care facility nearby, as trauma has been associated with high proportion of male ED use. As noted by Nicol et al., males account for 71.3% of traumatic injuries.^12^ The male predominance in ED use is reflected in other studies. Two teams of researchers across the Middle East reported similar findings of male predominance of 51.2% and 55%.^13,14^ A systematic review of similar facilities in sub-Saharan Africa indicates that there are generally more male ED users (55.7%; IQR: 50.0% – 59.2%).^9^ The male predominance observed in certain regions may also be because of sociocultural factors such as religion, which have been found to impact access to healthcare by females in Moslem countries.^15^ However, an older study from the same region alluded to greater parity in terms of the male/female distribution of ED users.^10^

The results of our study indicate that most ED users aged 15–64 years (62.7%) were unemployed as indicated in Table 1. Some studies reported an association between low socioeconomic conditions and frequent, inappropriate ED use.^16,17,18^ Furthermore, formally employed patients have active, employer-subsidised medical insurance that allows them to access private healthcare explaining the disproportionate representation of the employed.

One-third of ED users at BDH reside within 5 km of the hospital, with progressive reduction in the proportion of users as distance from the hospital increases. It is possible that this pattern would have been more pronounced but for the use of the EMRS as a transportation mode. Regardless of 60% of ED users being self-referred, inappropriate ED use at BDH remains doubtful as only 6% of these patients presented with ‘routine’ conditions.

This study reveals that the incidence of self-referral is high among ED users at BDH. Sixty percent of patients visiting the ED were self-referred. Only 37.8% of these patients were appropriately referred from other healthcare facilities. A study at Paarl Hospital in Cape Town, South Africa, demonstrated a higher proportion of self-referral (88.2%) among ED users.^19^ The proportion of ‘routine’ patients accessing medical services at the ED was quite low (5.7%) as compared to findings of a study that showed a far greater proportion of ‘routine’ ED users (65%) at George Provincial Hospital in Western Cape Province.^20^ Most patients seen at the ED in BDH were triaged ‘urgent’ (68.2%). The highest proportion of self-referred patients by level of acuity was found in the ‘routine’ triage category (65%) and the proportion of self-referrals increased as the level of acuity of the patient’s condition decrease.

The most frequent diagnostic classifications from the results of this study include ‘trauma-related conditions’ (36.5%), respiratory conditions (9.5%), gastrointestinal illnesses (9.5%), genitourinary conditions (8.4%), ‘ENT’ (5.3%) and ‘poisoning and intoxication’ (5.1%). However, a study conducted at Kenyatta National Hospital in Nairobi, Kenya, found ‘non-communicable diseases’ (35%) more common than trauma-related conditions (24%).^21^ Infections constituted the commonest diagnostic classification at the Princess Marina Hospital, Gaborone, Botswana, making up over a quarter (25.6%) of cases seen at the ED.^8^ Meanwhile, at the National District Hospital, Bloemfontein, South Africa, ‘HIV-related complications’ was the commonest diagnosis, accounting for a fifth of cases seen.^11^ The ED caters for a heavy burden of trauma-related conditions, making them a leading presentation and diagnostic classification at BDH ED.

The emergency department length of stay (EDLOS) at BDH varies greatly among patients. However, about half of them will spend less than 3 h as EDLOS. The ED at BDH seems capacitated to handle the wide variety of conditions seen at the unit as most of the ED users are treated and discharged from the hospital following consultation (75%). Only 14.4% of these ED users were admitted into the wards after ED consultation and treatment. The proportion of ED users referred to level two and academic hospitals for specialised care was 6.5%. Sporadically, patients report inappropriately at the ED (1%) and are consequently redirected to lower levels of care such as clinics. This might indicate that users of the ED do so appropriately. Moreover, the decision to down-refer remains a difficult one for facility managers as some of the patients may later re-present at the ED with complications. Also, the SATS tool, under ideal use, has been shown to have a 15% likelihood of overtriage, indicating that recorded triage scores might be exaggerated.^22^

Findings of this study contribute to the literature and knowledge to assist policy makers, health facility managers and public health practitioners to define and strengthen emergency services. It is also anticipated that a national and global audience responsible for the promotion of emergency care at the primary care level in developing countries will find relevance with the results of the study.

This study had limitations of having been conducted for a limited time at only one district hospital that may not be representative of similar level facilities in the developing or underdeveloped countries.

## Conclusion

This study found a high utilisation of the ED by self-referred ED users (60%) and for trauma-related cases. In spite of these, the ED at BDH is mostly appropriately used, as only 5.6% of the patients came with routine conditions. It is recommended that local public health authorities address the burden of trauma-related cases through identification and mitigation of modifiable risk factors/behaviour within the community.
